# *In Vivo* Comparison of Two Human Norovirus Surrogates for Testing Ethanol-Based Handrubs: The Mouse Chasing the Cat!

**DOI:** 10.1371/journal.pone.0017340

**Published:** 2011-02-24

**Authors:** Syed A. Sattar, Mohammad Ali, Jason A. Tetro

**Affiliations:** Center for Research on Environmental Microbiology (CREM), University of Ottawa, Ottawa, Ontario, Canada; The University of Hong Kong, Hong Kong

## Abstract

Human noroviruses (HuNoV), a major cause of acute gastroenteritis worldwide, cannot be readily cultured in the lab. Therefore, a feline calicivirus (FCV) is often used as its surrogate to, among other things, test alcohol-based handrubs (ABHR). The more recent laboratory culture of a mouse norovirus (MNV) provides an alternative. While MNV is closer to HuNoV in several respects, to date, no comparative testing of FCV and MNV survival and inactivation on human hands has been performed. This study was designed to address the knowledge gap. The rates of loss in viability during drying on hands were −1.91 and −1.65% per minute for FCV and MNV, respectively. When the contaminated skin was exposed for 20 s to either a commercial ABHR with 62% (v/v) ethanol or to 75% (v/v) ethanol in water, FCV infectivity was reduced by <1 log_10_ while that of MNV by nearly 2.8 log_10_. Extending the contact time to 30 s reduced the FCV titer by almost 2 log_10_ by both test substances and that of MNV by >3.5 log_10_ by the commercial ABHR while 75% ethanol did not show any noticeable improvement in activity as compared to the 20 s contact. An 80% (v/v) aqueous solution of ethanol gave only a 1.75 log_10_ reduction in MNV activity after 20 s. The results show significant differences in the ethanol susceptibility of FCV and MNV in contact times relevant to field use of ABHR and also that 62% ethanol was a more effective virucide than either 75% or 80% ethanol. These findings indicate the need for a review of the continuing use of FCV as a surrogate for HuNoV.

## Introduction

Human noroviruses (HuNoV) remain the most common cause of acute viral gastroenteritis (AVG) with 6 million clinical cases and 200,000 deaths/year worldwide [1]. Outbreaks of HuNoV are frequent in healthcare [2], community settings [3] and onboard cruise ships [4]; nosocomial outbreaks can close hospital wards [5]. The virus is shed in feces and in vomitus [6] and is relatively stable outside hosts [7], [8].

Since laboratory culture of HuNoV is difficult, the feline calicivirus (FCV) is often used as its surrogate in testing, among other things, microbicides [9], [10]. However, FCV may be unsuitable for this purpose due its higher acid sensitivity and also because it is normally a respiratory pathogen in felines [11].

The successful *in vitro* culture of a murine norovirus (MNV) now provides an alternative to FCV [12]; MNV is similar in acid resistance to HuNoV and also causes in mice a disease highly reminiscent of AVG in humans [12]. These factors, and a closer genetic kinship to HuNoV, could make MNV a better surrogate for testing microbicides, including alcohol-based handrubs (ABHR).

Comparative studies between MNV and FCV have been published; however, they have focused either on the environmental survival of the viruses [11], [13] or compared the activity of chemicals against FCV and MNV in *in vitro* settings [14]–[16]. There has been no documented comparison of the activity of ethanol and ethanol-based ABHR against FCV and MNV using an *in vivo* test protocol. The information gained from such a comparison would allow for a better understanding of the choice of surrogate for HuNoV. This study was designed to fill the knowledge gap.

## Materials and Methods

### ABHR tested

The concentrations of ethanol used are given as volume/volume (v/v). The ABHR tested were one commercial gel with 62% ethanol as the only listed active, and aqueous ethanol solutions at 75% and 80%.

### Subjects

This study was approved by the Ottawa Hospital Research Ethics Board (OHREB) under protocol #2000289-01H: The use of Fingerpads of Adult Volunteers to Investigate the Germicidal Activity of Handwash and Handrub Agents. Persons (20–60 years in age), were briefed on the project and those willing to participate gave signed consent. Each subject then received microbicide-free personal care items for use starting a one week before the first day of participation in the study. Six subjects were used in each comparative test.

### Viruses

Strain F9 of FCV (ATCC #VR-782) was received from Dr. S. Bidawid (Health Canada, Ottawa, ON) and MNV Type 1 was a gift from Dr. H.W. Virgin (Washington Univ., St. Louis, MO).

### Host cells

CrFK cells (ATCC CCL-94) and RAW 267.4 cells (ATCC #TIB-71) were also obtained from Dr. Bidawid and were used to grow and plaque assay FCV and MNV, respectively.

### Preparation of virus pools

Monolayers of both types of cells were grown at 36±1°C in Eagle's minimal essential medium (MEM; Invitrogen, Burlington, ON, Canada) with 10% fetal bovine serum (FBS; Invitrogen Burlington, ON, Canada), 2% L-Glutamine (Invitrogen), and 2% of 7.5% sodium bicarbonate (NaHCO_3_.7H_2_O) in 75 cm^2^ plastic flasks (Sarstedt, Saint-Leonard, QC, Canada).

Each monolayer separately received 200 µL of FCV or MNV and the flasks were held for one h at 36±1°C for virus adsorption. Ten mL of MEM supplemented with 2% FBS was added to each flask and reincubated at 36±1°C till nearly 80% of each monolayer showed virus-induced cytopathology. The flasks were then subjected to three freeze-thaw cycles for virus release, their contents centrifuged at 1,000×*g* for ten minutes and the supernatant was pooled and then aliquoted for storage at −80°C.

### Soil load

All virus inocula contained a soil load to simulate the presence of feces or vomitus. A 340-µl volume of virus suspension was mixed with 35 µl 5% Tryptone (BD Difco; Mississauga, ON, Canada), 25 µl of 5% bovine serum albumin (BD Difco; Mississauga, ON, Canada), and 100 µl of 0.4% mucin (type 1) from bovine submaxillary glands (Sigma, St. Louis, MO), all in sterile normal saline in phosphate buffer (pH 7.2) with a total protein content roughly equal to that in 5% FBS [17]. The final concentration of the virus was approximately 10^7^ plaque-forming units (PFU)/mL.

### Plaque assays

The plaque assay methods for FCV and MNV were as described previously by Wobus et al. [18] and Bidawid et al [12], respectively. Briefly, host cell monolayers were prepared in 12-well cluster plates (Corning; Fisher, Ottawa, ON, Canada) in 2 mL of growth medium (supplemented MEM with 10% FBS) in each well and incubated at 36±1°C in a 5% CO_2_ atmosphere for 16–18 h. The medium was aspirated and 0.1 mL of the virus sample diluted in Earle's Balanced Salt Solution (EBSS; Invitrogen) was placed separately in each of at least three wells. Each plate also included two wells as negative controls (0.1 mL EBSS only) and one well as positive control (0.1 mL of undiluted virus). The inoculated plates were incubated one h for virus adsorption. Each well then received 2 mL of an agar overlay and reincubated for plaque formation for 18 and 36 hours for FCV and MNV, respectively. The monolayers were then fixed and stained for PFU as described [18].

### Inoculating fingerpads

The subject's hands were first inspected to ensure freedom from any apparent damage. The subject then removed all ornaments from the hands and forearms and washed the hands with a non-medicated soap, rinsed them with running tap water (∼40°C) and dried them with a paper towel. Five mL of 70% ethanol was put into the subject's cupped hands for rubbing over both hands till dry. The pad of each digit was pressed against the mouth of an empty 2.0 mL cryovial (Sarstedt, St. Leonard, QC, Canada) for a circular indentation to place the inoculum at its centre. The subject then sat with the hands inside a laminar-flow hood to receive the virus. A virus inoculum of 10 µl was placed on each fingerpad using a positive-displacement pipette.

The air temperature and relatively humidity in the lab during the experiments ranged between 22–26°C and 45–55%, respectively.

### Virus elution

The digit to be eluted was placed over the mouth of a cryovial containing 1 mL of the eluent consisting of EBSS supplemented with 0.1% Tween-80 (Sigma-Aldrich, Oakville, ON, Canada), which neutralized the test solution through dilution and disaggregated viral clumps to provide a more realistic assessment of inactivation. The vial was inverted with the digit still in place and the eluent brought into contact with the contaminated area for 10 seconds followed by 20 full inversions. The 10-second contact and the inversion steps were performed once more. The vial was then turned upright and the surface of the skin was firmly scraped against the inside rim of the vial in a downward motion. Each fingerpad was inspected to ensure that the skin was free of any visible liquid. The skin of each pad was decontaminated by pressing it against a paper towel soaked in domestic bleach (1,000 ppm available chlorine) for 3 minutes. The subject then washed both hands with plain soap and dried them thoroughly before leaving the lab.

### Testing virus survival

Both thumbpads received 10 µL of the test virus in soil load and the inocula eluted immediately representing the ‘zero-time’ control or the number of PFU placed on each digit. Each fingerpad then received 10 µL of the virus and observed for drying. The inocula were eluted from two randomly-selected fingerpads (one left and one right) simultaneously after 20, 30 and 40 min.

### Virucidal activity of test formulations and controls

This procedure was based on the standard #E-1838-10 of ASTM International [17]. Briefly, both thumbpads received 10 µL of the test virus and the inoculum was eluted immediately and simultaneously from both (see below) and the thumbpads decontaminated; this represented the ‘zero-time’ control or the number of PFU placed on each digit. Each fingerpad then received 10 µL of virus and the subject kept the hands still in a laminar flow hood with the blower on till the inocula were visibly dry. Two randomly-selected fingerpads were eluted at this stage for the ‘baseline value’ or the PFU that survived the drying.

To test for virucidal activity, at least two randomly-selected fingerpads (one from each hand) were exposed simultaneously to 1.0 mL of the test substance or control fluid in a 2-mL cryovial by placing each fingerpad separately over the mouth of a vial and inverting to bring the fluid into contact with the skin for the required contact time. The fingerpad was then eluted as described above but the liquid remaining on the skin was not scraped off. The virus inoculation, drying, exposure and elution steps were repeated for the remaining fingers. The fingerpads were decontaminated with diluted bleach and washed as mentioned above.

### Data Analysis

For virus survival, regression analysis was performed to determine a rate of decay for each virus. Rates of decay were compared between viruses using a one-way Analysis of Co-Variance (ANCOVA). For virus inactivation experiments, six replicates were performed for each inactivation test and statistically analyzed using the Analysis of Variance (ANOVA) test with post-hoc Tukey Honestly Significant Difference (HSD) analysis.

## Results

### Virus survival on hands

**Figure 1:**
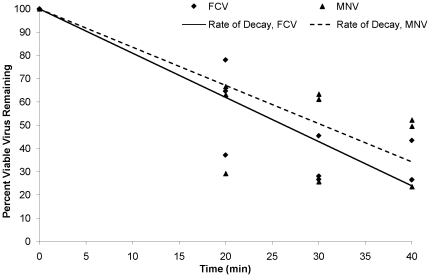
Survival of FCV (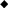) and MNV (▪) on human skin. Ten µL of virus in soil load was placed on the fingerpads of volunteers and allowed to dry. After 20, 30 or 40 minutes, the virus was eluted off the fingerpads and enumerated using plaque assays. Lines of decay were calculated using regression analysis and determined to be 1.91 percent per minute for FCV (r^2^ = 0.82) and 1.65 percent per minute for MNV (r^2^ = 0.64). Analysis of Co-Variance (ANCOVA) testing of the two regression lines demonstrated no significant difference between the two viruses (F = 0.61; p = 0.44).

Survival of the viruses on the fingerpads was normalized to a percentage survival to demonstrate the comparative decay in the viability of the viruses. Three samples for each time point and each virus were analyzed to ensure proper regression and ANCOVA analysis could be performed. As shown in Figure 1, virus survival was negatively associated with time. FCV viability was reduced at a rate of 1.91 percent per minute (r^2^ = 0.82). MNV reduction was 1.65 percent per minute (r^2^ = 0.64). Using the one-way ANCOVA test, the two rates of decay were not found to be significantly different (F = 0.61; p = 0.44).

### Inactivation of FCV and MNV by ethanol-based ABHR and Ethanol

**Figure 2:**
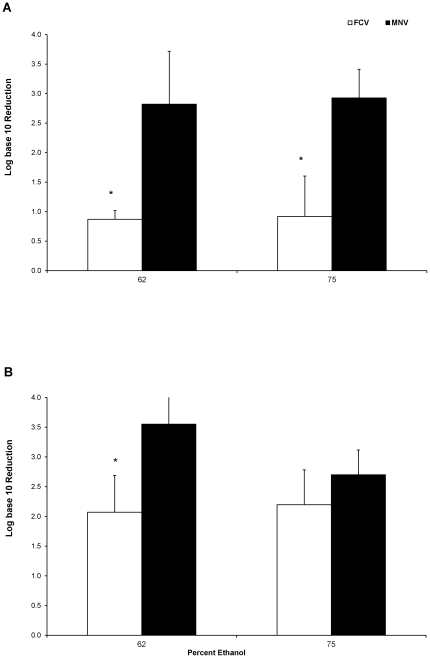
Reduction in the viability of FCV and MNV after contact with ethanol. Virus reduction was observed after 20 s (A) or 30 s (B) contact time with a commercial ABHR (62% ethanol) and an aqueous solution of 75% (v/v) ethanol on the fingerpads of adult subjects (n = 6). Significant difference between FCV and MNV (p<0.01) indicated by asterisks.

Six subjects for each concentration and contact time were used to allow for proper statistical analyses. Fig. 2A and 2B show the reduction in the viability of both viruses after 20 s and 30 s of contact, respectively. At 20 s, both levels of ethanol produced a<1 log_10_ reduction in FCV infectivity, while the reductions in MNV infectivity was almost 3 log_10_. Increasing the contact time to 30 s enhanced the reduction in FCV infectivity to about 2 log_10_ irrespective of the ethanol concentration. Whereas there appeared to be no significant difference between the activities of 62% and 75% ethanol against MNV at 20 s (Figure 2A), a difference was noted between the activities of the two ethanol concentrations with a contact time of 30 s (Figure 2B). However, the results of ANOVA and Tukey analyses were inconclusive due to variations in the data.

**Figure 3:**
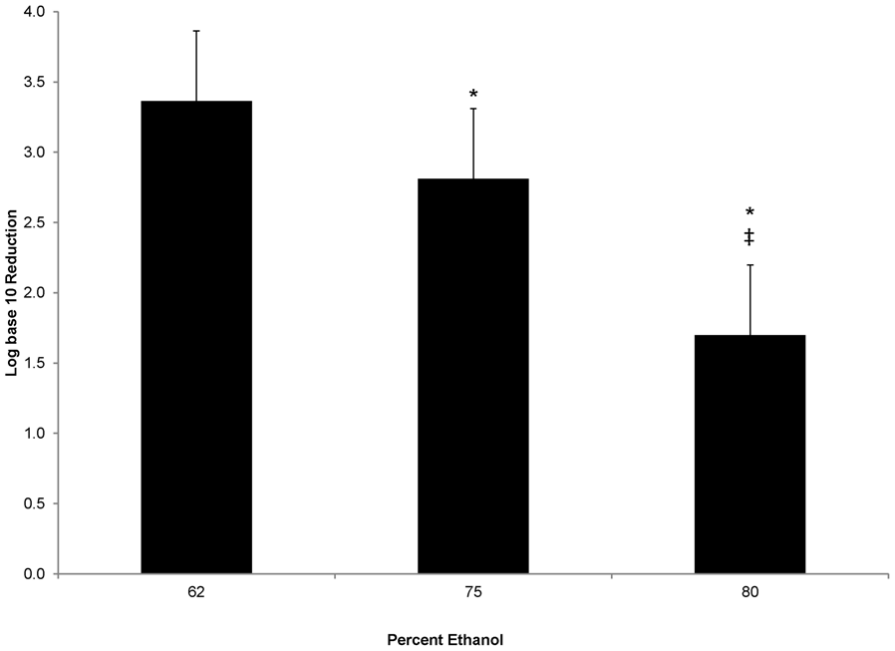
Reduction of MNV after 20 second contact with ethanol. MNV reduction was observed after 20 s contact time with one of either a commercial ABHR (62% ethanol), 75% ethanol or 80% aqueous ethanol solution on the fingerpads of adult subjects (n = 6). Significant differences were observed between both 62% and 75% (p<0.01) and 80% (p<0.01) indicated by asterisks (*). A significant difference was also seen between 75% and 80% (p<0.01) indicated by the double dagger (‡).

We thus compared the activities of three concentrations of ethanol against MNV at a contact time of 20 seconds. The results (Fig. 3) showed 62% ethanol was better than 75% ethanol (p<0.01 ANOVA; p<0.01 Tukey HSD) and that both concentrations showed better activity than the 80% solution (p<0.01 ANOVA; p<0.01 Tukey HSD).

## Discussion

This is the first report on comparative testing of the survival on skin and ethanol resistance of the two leading surrogates for HuNoV under conditions that closely mimic actual field use.

Our findings suggest that, the ability of the two viruses to survive on the hands of adults over the normal drying time [19]) was not significantly different. However, the time-associated reduction did demonstrate the necessity to perform reduction calculations using the baseline value (i.e. dried virus) as the starting point rather than the zero time.

The commercial product chosen claimed only one active ingredient, ethanol, with no other antimicrobial activity from the excipients. Other commercial products with higher concentrations of alcohol were found to be a mixture of ethanol and isopropanol, which was outside the scope of this study. Moreover, several formulations contained other excipients such as organic acids, which could bias the study and thus were not included. The choice of an aqueous ethanol solution was to ensure that only ethanol was the active to be tested. It should be noted here that non-alcohol-based formulations usually also are aqueous with only one active ingredient. However, the actives are often a quaternary-ammonium compounds (quats), which are weak in their activity against non-enveloped viruses in particular.

There have been several studies focusing on the resistance of FCV or MNV to various alcohol solutions [10], [20], [21]. However, there has been little consistency between the methods, the test materials, the viral challenge and the contact time. This has unfortunately led to confusion with respect to the use of ABHR and a plethora of comments and claims of activity that may not be entirely accurate for field use. The *in vivo* method used in this study is a standard of ASTM International [17] and it is recommended by the World Health Organization [22] for testing ABHR against human pathogenic viruses. It has been used previously for studies focusing on the *in vivo* evaluation of virucidal activity of antiseptics [19], [23]–[25]. The test gives data comparable to that with the whole-hand method [19], [24], while permitting a more thorough inspection of the hands of the subjects for any damage, thus making their experimental contamination virtually risk-free. Further, much smaller volumes of virus pools are required for the fingerpad method while allowing for replicates and controls being tested on the same subject in any given sitting. Perhaps the most useful aspect of this test is the ability to control the contact time precisely. In this study, contact times were at their lowest 20 seconds, which is perhaps the shortest time tested yet may be the most accurately representative of field use [20].

Since ABHR are no-rinse formulations, they are expected to inactivate pathogens *in situ* to achieve hand antisepsis. In view of this, the test substance remaining on the fingerpads at the end of the contact time was not scraped off but allowed to remain there for subsequent elution to better represent field use.

Even though ABHR are meant for use on hands free of visible contamination, a certain level of soiling of hands is inevitable during casual contact with the environment. The soil load added to the virus inocula was to represent this low level of organic and inorganic contamination on the skin, the potential protection it could afford to handborne pathogens and the ability of the tested formulations to achieve virus inactivation in its presence.

The observed differences between the susceptibilities of the two viruses to ABHR in general may be due to the structural properties of the viral capsid. Ethanol is well known for its ability to destabilize water and hydrophilic amino acids [26]. The cell receptor binding motif of FCV consists of a hypervariable region with mainly hydrophobic, neutral residues [27]. The effect of ethanol concentration, therefore, would be minimal on the active site yet, over time, general capsid instability would occur, leading to inactivation. The findings of this study correlate well with those of Kampf *et al*. who showed no significant difference in FCV virucidal activity with varying concentrations of ethanol between 75 and 95% [9]. In contrast to FCV, the cell receptor binding motif of MNV consists of numerous hydrophilic residues, which would be affected by concentrations of ethanol ≥45%. In this study, the 62% ABHR reduced the MNV titre by nearly 2.5 log_10_ (99.7%) in as little as 15 seconds and by 20 seconds, the titre was reduced by over 3.0 log_10_ (>99.9%).

Of particular note is the lowering of virucidal activity with the increase in ethanol concentration (Fig. 2 & 3). Parke and Birch [28] investigated various concentrations of ethanol and found that optimal destabilization of water molecules (and thus hydrophilic amino acid residues) did not positively correlate with higher ethanol concentrations but peaked at or near 62% ethanol and lessened exponentially as the concentration increased. Thus, the effect of 75% or 80% ethanol would be significantly less than that of 62% ethanol at shorter periods of time. The data presented here follows with that of Gehrke et al [21] who demonstrated a similar difference of efficacy between 62% and higher concentrations of ethanol (as well as 1- and 2-propanol) with longer contact times of 0.5 and 1.0 minutes.

One limitation of this study was that the 62%-ethanol-based test substance was a commercial ABHR while the 75% and 80% solutions were non-commercial. However, as discussed above, the presented data focused on ethanol alone as the active ingredient and thus should not be extrapolated to other formulations that contain isopropanol, organic acids or other residual antimicrobial agents. That being stated, the dramatic difference in efficacy as it relates to contact time (Fig. 3) should not be overlooked.

The comparative data presented here using a standardized *in vivo* test protocol shows that MNV, a virus much closer to HuNoV in several respects, is more readily inactivated by ABHR in relatively short contact times. This suggests the need for a wider acceptance of MNV as a surrogate for HuNoV and eventual phase-out of FCV for the purpose. Health Canada's recent guidance document on human-use antiseptic drugs now recommends the use of MNV as a surrogate for HuNoV to generate data for product registration [29]. Any further discussion of the topic of surrogates in this regard should wait till one is able to routinely culture and assay the infectivity of HuNoV *in vitro*. Such a change may also help reverse the impression in the minds of many infection preventionists that ABHR are ineffective in dealing with HuNoV outbreaks, based on the available data using FCV as its surrogate.

This comparative *in vivo* study showed that at shorter contact times, MNV is less resistant to ethanol-based ABHR than FCV. Extending the contact time reduced the difference between the two viruses with 75% ethanol but not with 62% ethanol. These differences may be a result of the various physicochemical characteristics of FCV and MNV but that ethanol concentration and contact time do play a significant role in the performance of an ethanol-based ABHR.

## References

[1] Patel MM, Widdowson MA, Glass RI, Akazawa K, Vinje J (2008). Systematic literature review of role of noroviruses in sporadic gastroenteritis.. Emerging Infectious Diseases.

[2] Koopmans M (2009). Noroviruses in healthcare settings: a challenging problem.. Journal of Hospital Infection.

[3] Gould D (2009). Management of Norovirus gastroenteritis in the community.. Br J Community Nurs.

[4] Vivancos R, Keenan A, Sopwith W, Quigley C, Mutton K (2010). Management of an international outbreak of norovirus on board a cruise ship.. International Journal of Infectious Diseases.

[5] Hansen S, Stamm-Balderjahn S, Zuschneid I, Behnke M, Rüden H (2007). Closure of medical departments during nosocomial outbreaks: data from a systematic analysis of the literature.. Journal of Hospital Infection.

[6] Kimura H (2010). A norovirus outbreak associated with environmental contamination at a hotel.. Epidemiol Infect.

[7] Lamhoujeb S, Fliss I, Ngazoa SE, Jean J (2008). Evaluation of the persistence of infectious human noroviruses on food surfaces by using real-time nucleic acid sequence-based amplification.. Applied and Environmental Microbiology.

[8] D'Souza DH, Sair A, Williams K, Papafragkou E, Jean J (2006). Persistence of caliciviruses on environmental surfaces and their transfer to food.. International Journal of Food Microbiology.

[9] Kampf G, Grotheer D, Steinmann J (2005). Efficacy of three ethanol-based hand rubs against feline calicivirus, a surrogate virus for norovirus.. Journal of Hospital Infection.

[10] Whitehead K, McCue KA (2010). Virucidal efficacy of disinfectant actives against feline calicivirus, a surrogate forÂ norovirus, in a short contact time.. American Journal of Infection Control.

[11] Cannon JL, Papafragkou E, Park GW, Osborne J, Jaykus LA (2006). Surrogates for the study of norovirus stability and inactivation in the environment: A comparison of murine norovirus and feline calicivirus.. Journal of Food Protection.

[12] Wobus CE, Karst SM, Thackray LB, Chang KO, Sosnovtsev SV (2004). Replication of Norovirus in cell culture reveals a tropism for dendritic cells and macrophages.. PLoS Biology.

[13] Bae J, Schwab KJ (2008). Evaluation of murine norovirus, feline calicivirus, poliovirus, and MS2 as surrogates for human norovirus in a model of viral persistence in surface water and groundwater.. Applied and Environmental Microbiology.

[14] D'Souza DH, Su XW (2010). Efficacy of Chemical Treatments Against Murine Norovirus, Feline Calicivirus, and MS2 Bacteriophage.. Foodborne Pathogens and Disease.

[15] Beekes M, Lemmer K, Thomzig A, Joncic M, Tintelnot K (2010). Fast, broad-range disinfection of bacteria, fungi, viruses and prions.. Journal of General Virology.

[16] Su XW, Howell AB, D'Souza DH (2010). The effect of cranberry juice and cranberry proanthocyanidins on the infectivity of human enteric viral surrogates.. Food Microbiology.

[17] ASTM International (2010). ASTM E1838 - 10 Standard Test Method for Determining the Virus-Eliminating Effectiveness of Hygienic Handwash and Handrub Agents Using the Fingerpads of Adults..

[18] Bidawid S, Malik N, Adegbunrin O, Sattar SA, Farber JM (2003). A feline kidney cell line-based plaque assay for feline calicivirus, a surrogate for Norwalk virus.. Journal of Virological Methods.

[19] Sattar SA, Ansari SA (2002). The fingerpad protocol to assess hygienic hand antiseptics against viruses.. Journal of Virological Methods.

[20] Cheeseman KE, Denyer SP, Hosein IK, Williams GJ, Maillard JY (2009). Evaluation of the bactericidal efficacy of three different alcohol hand rubs against 57 clinical isolates of S-aureus.. Journal of Hospital Infection.

[21] Gehrke C, Steinmann J, Goroncy-Bermes P (2004). Inactivation of feline calicivirus, a surrogate of norovirus (formerly Norwalk-like viruses), by different types of alcohol in vitro and in vivo.. Journal of Hospital Infection.

[22] World Health Organization (2010). WHO Guidelines on Hand Hygiene in Health Care.. http://whqlibdoc.who.int/publications/2009/9789241597906_eng.pdf.

[23] Sattar SA, Abebe M, Bueti AJ, Jampani H, Newman J (2000). Activity of an alcohol-based hand gel against human adeno-, rhino-, and rotaviruses using the fingerpad method.. Infection Control and Hospital Epidemiology.

[24] Steinmann J, Nehrkorn R, Meyer A, Becker K (1995). Two in-vivo protocols for testing virucidal efficacy of handwashing and hand disinfection. Zentralblatt fur Hygiene und Umweltmedizin  =. International journal of hygiene and environmental medicine.

[25] Bidawid S, Malik N, Adegbunrin O, Sattar SA, Farber JM (2004). Norovirus cross-contamination during food handling and interruption of virus transfer by hand antisepsis: Experiments with feline calicivirus as a surrogate.. Journal of Food Protection.

[26] Herskovits TT, Gadegbeku B, Jaillet H (1970). On the structural stability and solvent denaturation of proteins. I. Denaturation by the alcohols and glycols.. Journal of Biological Chemistry.

[27] Geissler K, Schneider K, Truyen U (2002). Mapping neutralizing and non-neutralizing epitopes on the capsid protein of feline calicivirus.. Journal of veterinary medicine B, Infectious diseases and veterinary public health.

[28] Parke SA, Birch GG (1999). Solution properties of ethanol in water.. Food Chemistry.

[29] Health Canada (2009). Guidance Document - Human-Use Antiseptic Drugs.. http://www.hc-sc.gc.ca/dhp-mps/prodpharma/applic-demande/guide-ld/antiseptic_guide_ld-eng.php.

